# Modelling CD4 counts before and after HAART for HIV infected patients in KwaZulu-Natal South Africa

**DOI:** 10.4314/ahs.v20i4.7

**Published:** 2020-12

**Authors:** Ashenfai A Yirga, Sileshi F Melesse, Henry G Mwambi, Dawit G Ayele

**Affiliations:** 1 School of Mathematics, Statistics and Computer Science, University of KwaZulu-Natal, Pietermaritzburg, Private Bag X01, Scottsville, 3209, South Africa; 2 Institute of Human Virology, University of Maryland, School of Medicine, USA

**Keywords:** Random-effects model, spatial covariance structure, CD4+ count, HAART, CAPRISA

## Abstract

**Background:**

This study aims to make use of a longitudinal data modelling approach to analyze data on the number of CD4+cell counts measured repeatedly in HIV-1 Subtype C infected women enrolled in the Acute Infection Study of the Centre for the AIDS Programme of Research in South Africa.

**Methodology:**

This study uses data from the CAPRISA 002 Acute Infection Study, which was conducted in South Africa. This cohort study observed N=235 incident HIV-1 positive women whose disease biomarkers were measured repeatedly at least four times on each participant.

**Results:**

From the findings of this study, post-HAART initiation, baseline viral load, and the prevalence of obese nutrition status were found to be major significant factors on the prognosis CD4+ count of HIV-infected patients.

**Conclusion:**

Effective HAART initiation immediately after HIV exposure is necessary to suppress the increase of viral loads to induce potential ART benefits that accrue over time. The data showed evidence of strong individual-specific effects on the evolution of CD4+ counts. Effective monitoring and modelling of disease biomarkers are essential to help inform methods that can be put in place to suppress viral loads for maximum ART benefits that can be accrued over time at an individual level.

## Background

Multilevel data modelling allows to account for the correlation of measurements, and include variables measured at different levels as well as model the variation at different levels. Longitudinal data, or repeated measurements data is a specific form of multilevel data. In longitudinal studies, repeated observations are made on an individual on one or more outcomes, including covariate information at a baseline and over time. Measurements made on the same individual are likely to be more similar than measurements made on different individuals. Thus, observations on the same individual will not be independent. That is, repeated measurements on the same subjects are bound to be correlated 1–3.

Longitudinal data analysis is widely used for at least three reasons: to increase the sensitivity by making within-subject comparisons, to study changes over time, and to use subjects efficiently once they are enrolled in a study^4^–^6^. Repeated measurements can compensate for small sample sizes because an individual is observed more than once compared to a cross-sectional study. The need for the covariance structure of the observed data makes longitudinal data analysis more complex than standard linear regression. For the inference to be substantial, the covariance among repeated measures must be appropriately modeled. Although the covariance structure is not the prime interest of the study, it is essential for valid inference 7,8. Therefore, a lot of efforts are needed at the beginning of the statistical analysis to assess the covariance structure of the data. Traditional methods for longitudinal data such as Analysis of Variance (ANOVA) and Multivariate Analysis of Variance (MANOVA) are of limited use because of the restrictive assumptions concerning the variance-covariance structure of the repeated measures 9. For this reason, mixed-effects models have become popular for modelling longitudinal data. This statistical procedure also permits the estimation of variability in hierarchically structured data and examines the impacts of factors at distinctive levels 10,11. Since longitudinal studies are often faced with the incompleteness of the data due to partially observed subjects, the mixed-effects model is by its very nature able to deal with unbalanced data of this nature.

Thus, this study was conducted to review the general Linear Mixed Model approach that can be extended for multivariate longitudinal data by assuming appropriate random effects. This method has the benefit of having extra correlation evolving from the longitudinal data structure that can be modeled within the same framework. Therefore, the focus of this study is to adopt the mixed-effects model with appropriate random effects incorporated, including a flexible variance-covariance structure that gives the best fit as well as identifying whether specific clinical and sociodemographic factors present in the data (and their respective possible interactions) influenced CD4 count in a cohort of HIV-Infected Patients. The information and understanding of such factors are of epidemiological importance. The results will be beneficial in developing tools to support clinicians in the identification of factors related to HIV-Infected Patients. The results can be further used to shape communication and counseling strategies at the individual level before treatment initiation.

## Materials and methods

### Data source

This study uses data from the Centre for the AIDS Programme of Research in South Africa (CAPRISA) 002 Acute Infection Study. The study was conducted on HIV-infected women at the Doris Duke Medical Research Institute (DDMRI) at the Nelson R Mandela School of Medicine of the University of KwaZulu-Natal in Durban, South Africa. Between August 2004 and May 2005, CAPRISA initiated a cohort study enrolling high-risk HIV negative women to follow up. Women infected with HIV were recruited into the Acute Infection Study and then followed up closely to study disease progression and CD4/viral load evolution^12^–^14^. Once HIV-Infected women enrolled in the AI study, their CD4 cell count and viral load were measured and assessed regularly. When their CD4 cell count is less than or equal to 350 cells/mm^3^ for more than two consecutive visits between 6 months or if they were with AIDS-defining illness (WHO clinical stage 3–5), they would be referred to a public government clinic for ARV treatment. However, these patients would only start HAART once their CD4 cell count was less than 200 cells/mm^3^, according to the National Department of Health South Africa until 2015. With effect from the 1st January 2015, according to the National Department of Health, the criterion to start HIV patients on early initiation of ART was a CD4 cell count less than or equal to 500 cells/mm^3^ 32,33.

### Method

Mixed-effects modelling is an advanced and vital method in statistics. It is a well-known method; therefore, we summarize the key aspects of the model relevant to the current study. The literature on mixed models is ubiquitous, and some of it can be found in 2,3,5,6,9,11,15–18. The use of the mixed-effects model for longitudinal data helps to correctly account for the correlation of observations within a subject and also to quantify the heterogeneity between subjects due to unobserved factors. It is important that before its implementation, adequate sample size is determined based on prior information on the magnitude of the correlation and the planned number of observations per individual. By correctly estimating the sample size, we end up with correctly estimated standard errors (SEs), which will give reliable confidence intervals (CI) and p-values. We can use the mixed-effects model to account for variation at lower and higher levels of the design structure. Accounting for variation at a lower level gives us more power for estimation at a higher level 3. A mixed model is made up of fixed and random effects where the latter helps in accounting for correlation at a lower level within higher-level units. That is why mixed models are called “mixed” because the coefficients are a mix of fixed and random effects.

In more general terms, fixed effects or fixed factors are covariates that we anticipate will influence the outcome variable. They are what we call explanatory variables in a standard linear regression. For instance, in our case, we are interested in making conclusions about how the socio-economic, demographic, and treatment type (place of residence, baseline BMI, baseline viral load, age, education level, marital status, HAART initiation, etc.) impacts the CD4+ count of a patient. Therefore, these socio-economic, demographic, and treatment types are fixed effects, and CD4+ count of a patient is the response variable. Thus, a fixed-effect is the parameter of interest. The overall intercept is not the variable of interest, but of course, it is a fixed effect. In addition to the fixed effects, we also incorporate random effects in the mixed-effect model. Random effects are grouping factors for which we are attempting to control. A random intercept allows a different intercept for each subject. A random effect for a variable enables the effect of a variable on the outcome to differ between subjects. For example, a random effect could also be a random slope for a categorical variable. In general, in a mixed model, all of the variables of interest are added as fixed effects, but at least one and sometimes several of the fixed effects variables may also be added as random effects variables 19. Therefore, the idea is that the values of a given random effect in the sample are a random sample of all possible values in the broader population (e.g., people in the sample are a random sample of people in the population). Moreover, in longitudinal studies, time or a time-varying covariate X is often an explanatory variable of interest, and the associations between explanatory variables and responses may vary between subjects. A model that allows heterogeneity in the intercept and heterogeneity in the magnitude of the slope between subjects is referred to as the random intercept and slope model. The random intercept and slope model is given by

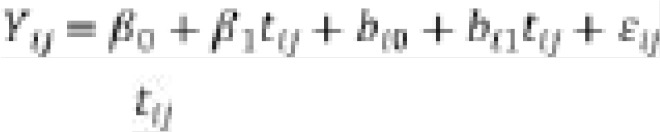

where *t_ij_* is the time variable used as a predictor in the model.

A more general form of the mixed model is expressed as

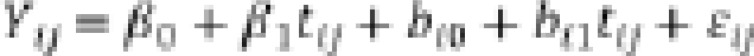

where *Y_ij_* is an outcome variable that indicates the *j^th^* measurement on the *j^th^* subject, *X_ij_, f* = 1,2,...,*p* are the predictor variables, *β*_0_, *β*_1_,...,*β*_y_ are fixed effects, *b*_11_,...,*b*_iy_ are random effects, and *ε_ij_*'s are residuals.

In the current model, the square root of CD4 count is used as the outcome because this transformation satisfies the normality assumption better than the untransformed CD4+ cell counts. Hence the model of interest is

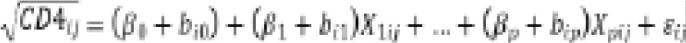
, where

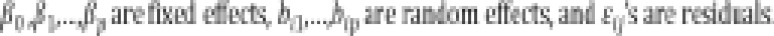


The general matrix specification of the mixed model is

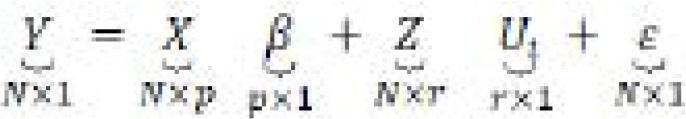

with *i* = 1, ..., *n* individuals and j = 1, ..., *N* observations for individual i. Thus, Y is a N × 1 vector of the response variable, *x* = [*x*_11_, ..., *x_ip_*] is N×*p* known design matrix that includes covariates for the fixed effects, β is p × 1 vector of fixed effects parameters, *Z* = [*X*_11_, ..., *X*_ir_] is N × r known design matrix for random effects, *U_i_* is *r* × 1 vector of random effects from a normal distribution with variance-covariance matrix G, and *ε* is N × 1 error vector from a normal distributionwith variance-covariance matrix R^19^.

Assumption: U and are independent and each is normally distributed.


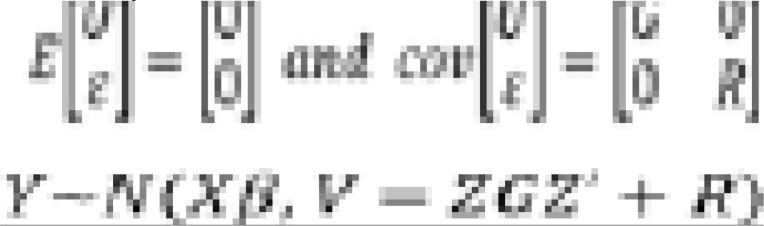

or

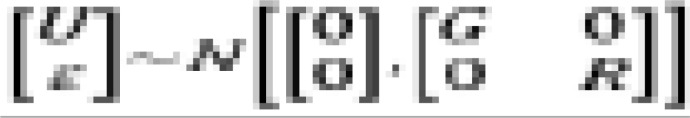


The distribution of Y is a multivariate normal distribution i.e. the vector of outcomes 
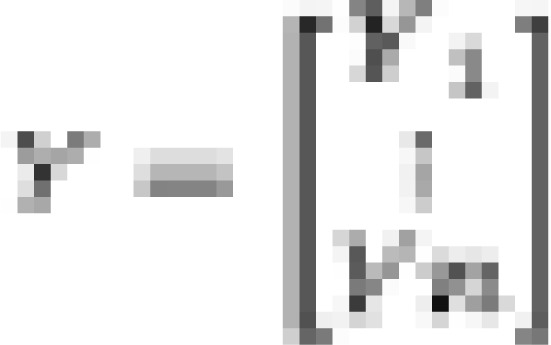
 is a multivariate normal distribution with mean vector *Xβ* and variance-covariance non-singular matrix V and its probability density function (pdf) is

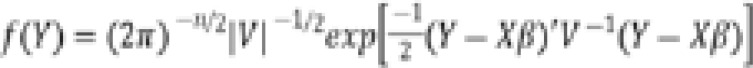


The log-likelihood of Y under this model is

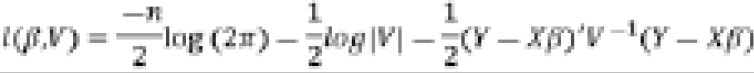

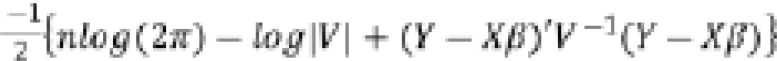


Therefore, the maximum likelihood estimate (MLE) of (*β, V*) is the one that maximizes the right-side of the above expression 19.

Covariance or correlation structures that are most commonly used for longitudinal data analysis are compound symmetry (CS), unstructured (UN), First-order Autoregressive (AR (1)), and Toeplitz (Toep). These four common covariance structures are summarized in 5,7,8,16,19–22. To decide which mixed-effects model fits the data best, we can use likelihood-based methods, i.e., either the likelihood ratio test (LRT) or Information Criteria (IC) such as Akaike Information Criteria (AIC) or Bayesian Information Criteria (BIC) method. The LRT, which is based on *γ*^2^-distribution can be used to test nested models. The model with the lowest AIC and BIC is the best fitting model. That is, the AIC and BIC can be used to compare models such that the smaller of any of these, the better between two or more competing models. The IC method is more general to compare two or more competing non-nested models. However, the LRT is the best method to compare nested models 23. In mixed-models, we use maximum likelihood (ML) to estimate the fixed effects, the standard errors of the fixed effects, and the variance of the random effects. The likelihood of mixed effect models can be time-consuming computationally, but with advances in statistical software, this has become an easily manageable problem. Often the likelihood is solved by iteration until convergence. However, under ML estimation the residual variance and variance of random effects are underestimated thus instead the restricted maximum likelihood (REML) estimation gives unbiased estimates of variance parameters by taking into account the degrees of freedom used to estimate the fixed effects hence variance parameter estimates are generally larger than those from ML estimation. However, REML uses the covariate mean structure (the number of fixed effects) in the model estimation steps. That means we use REML when we are comparing two models that differ only in random effects (see page 352 in Der and Everitt, 2012) 4,24.

In general, when testing mixed-effects models that differ in variance components, we could either use REML or ML since they both give interpretable LRT and IC for such a comparison. However, testing and comparing models that differ in fixed effects, then only ML, will provide us with interpretable LRT and IC. However, ML does not take into account the degrees of freedom for the loss of fit in the estimation of parameters, but REML does 19,20.

## Results

**Figure 1:**
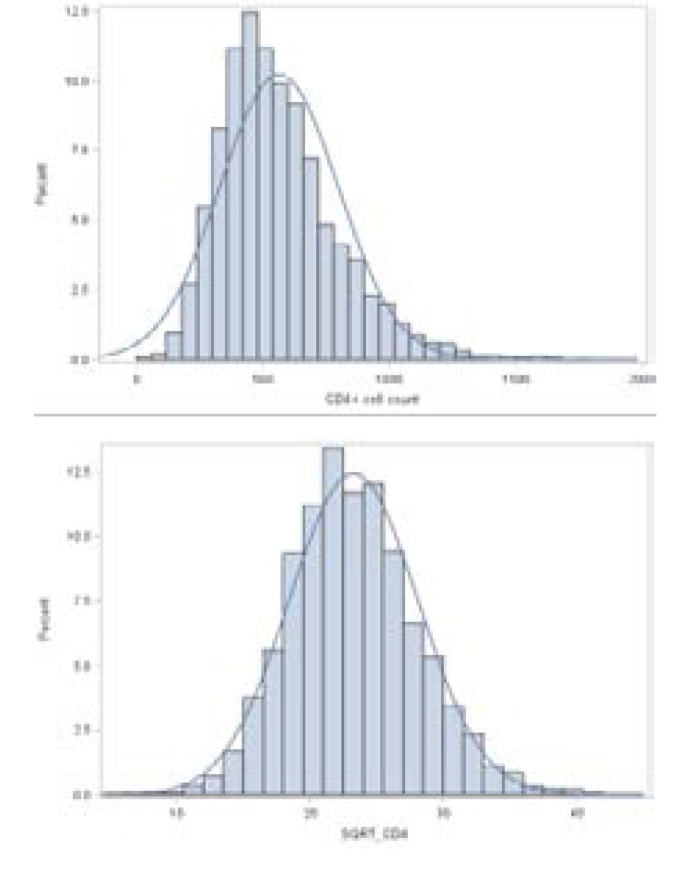
Distributional properties plot for original and square root transformed CD4 trajectories

Data for this study were obtained from the CAPRISA 002: Acute infection Study, which was initiated between August 2004 and May 2005^13^. The baseline characteristics of the datasets are given in Table 1. From a total of 235 women, 105 (44.7%) were residing around Vulindlela (rural site), and 130 (55.3%) were residing around eThekwini (Durban, urban site), KwaZulu-Natal, South Africa. The average age at enrollment and baseline CD4+ cell counts was 27.15 years (range 18–59) with a standard deviation of 6.56 and 570 (range 182- 1575) with a standard deviation of 229.6, respectively. The average follow-up time was 2.69 years, and the majority of the women 182 (77.4%) had a stable partnership. Furthermore, from the total women included in the study, the majority of the 224 (95.3%) completed secondary/high education, and most of the women (78.8%) were self-reported sex workers^13^,^34^. There were a total of 7129 observations from the 235 women, which consists of a minimum of four and a maximum of sixty-one measurements of CD4+ cell counts, among the subjects which were measured at different time points indicating that the number of measurements over all subjects was not equal. Further apart from an unequal number of measurements across individuals, measurements were not taken at fixed time points, which implies the CAPRISA 002: Acute Infection Study is a highly unbalanced longitudinal data set that requires carefully designed modelling approaches. Figure 1 (left panel) shows that CD4+ cell count distribution is right-skewed, indicating non-normality; thus, a square root transformation to CD4+ cell count was performed to normalize the data, Figure 1 (right panel) shows that the square root transformed data conforms quite well to the normal distribution.

**Table 1:** Baseline characteristics of the motivated data set (CAPRISA 002), 2004–2018

Variable	Total	Variable	Total
**Number of women**	235	**Marital Status**	
**Place of residence**		**No partner**	43 (18.3%)
Rural	105 (44.7%)	Stable partner	182 (77.4%)
Urban	130 (55.3%)	Many partners	10 (4.3%)
**Age at Seroconversion (Years)**			
Mean (Std. Deviation)	27.15 (6.56)	**Educational Attainment**	
<20	21 (8.9%)	Primary schools (grade 0–7)	11 (4.7%)
20–29	150 (63.8%)	Secondary schools (grade 8–12)	224 (95.3%)
30–39	50 (21.3%)	**Baseline CD4+ cell counts (cells/µL)**	
40–49	12 (5.1%)	Mean (Std. Deviation)	570 (229.6)
≥ 50	2 (0.9%)	**Baseline HIV viral load (cells/µL)**	
**Baseline Body Mass Index**		Undetectable VL (< 50)	1 (0.4%)
Underweight	14 (6%)	Low VL (50<VL<10000)	74 (31.5%)
Normal weight	173 (73.6%)	Medium VL (10000<VL<100000)	94 (40%)
Overweight	41 (17.4%)	High VL (≥100000)	66 (28.1%)
Obese	7 (3%)		

**Figure 2:**
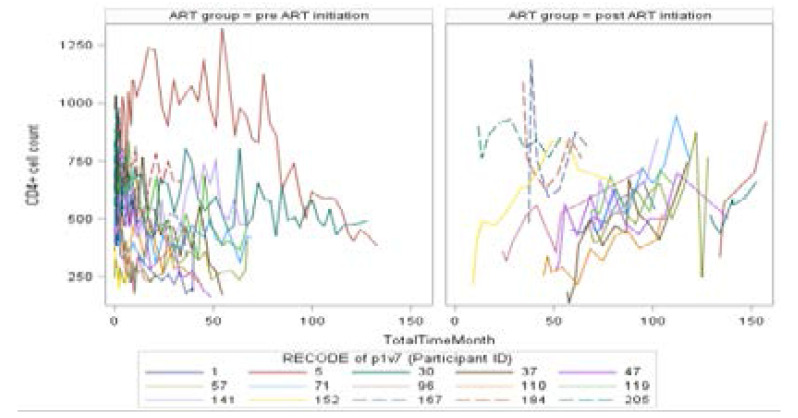
Individual profiles plot of CD4+ count for the same 15 randomly selected individuals before and after HAART.

The spaghetti plots in Figure 2 illustrate the actual CD4+ cell count measurements for randomly chosen participants over time across pre and post ART initiation groups. Since plots with all individual curves can be hard to distinguish for large sample size, we randomly chose 15 participants to construct such individual plots. From Figure 2, it can be seen that there is a decreasing trend of CD4+ cell count overtime on patients before Highly Active Antiretroviral Therapy (HAART) initiation, but an increasing trend of CD4+ cell count overtime for the same 15 randomly chosen patients initiated on HAART. Figure 2 also shows that there is evidence of variability between individuals as well as variability within individuals. Besides, the individual profiles are not all of the same lengths, an indication of incompleteness and missing data due to dropout or attrition.

**Figure 3:**
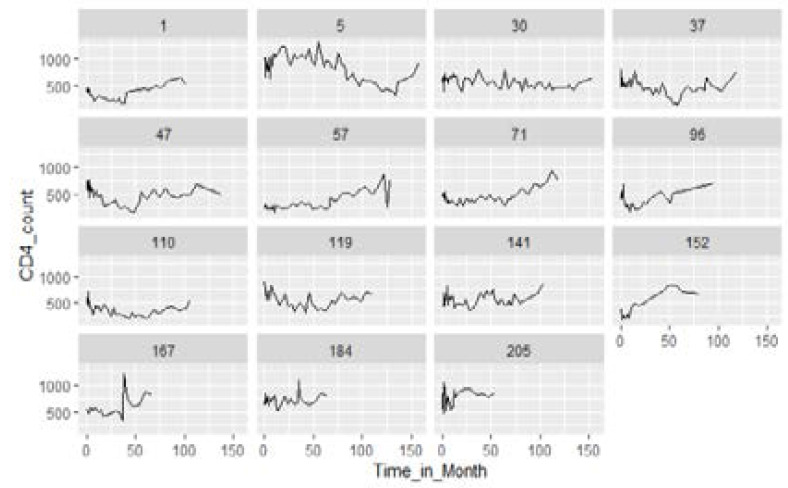
A sample of 15 individual CD4 trajectories versus time from the CAPRISA 002 AI Study

Figure 3 shows an array of individual series from the CAPRISA 002: AI study. In each panel, the observed CD4 count for a single subject is plotted against the times that measurements were obtained. Such plots permit assessment of the person response patterns and whether there is substantial heterogeneity within the trajectories. Figure 3 shows that there can be variation in the “level” of CD4 count for subjects. Subject PID=5 in the first row second from left has CD4 counts greater than 500 for almost all times while PID= 110 in the third row lower-left corner has all measurements below 500. Moreover, PID=30 in the first row third from left has all measurements almost constant around 500. Further, individuals profile plots can be evaluated for the change over time 6. Figure 3 shows that most subjects are either relatively stable in their measurements over time, or tend to be increasing.

**Figure 4:**
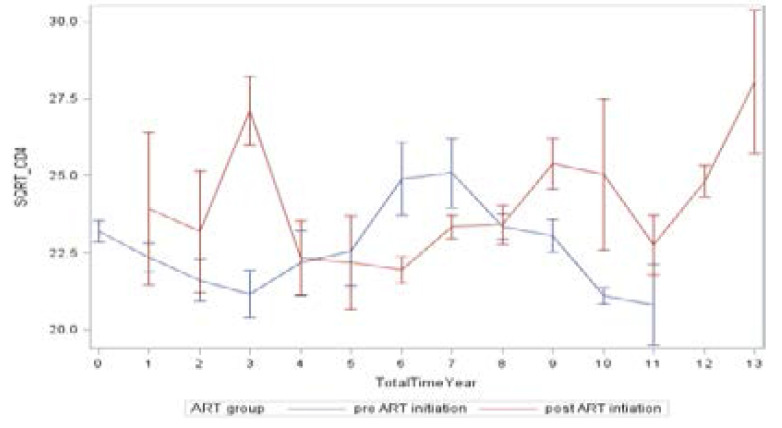
Mean CD4 trajectories over time by ART Initiation group, CAPRISA 002 AI study

Figure 4 shows the mean CD4 trajectories overtime for the pre and post ART initiation groups in the CARISA 002: AI study. Overall the mean plots suggest that patients initiated on HAART have significant quadratic growth in the evolution of CD4 count over time as what we would expect. Furthermore, the plots exhibit non-linearity implying factors that control the nonlinear effect that may need to be incorporated in the model. The inferential focus of this study is on the mean response of a square root transformation to CD4+ cell count measure. First, an appropriate selection of the random effects was also performed. That is the appraisal as to which of the nonlinear components (the intercept, time, or square root of time) ought to have a random component was made. To have a valid inference about the mean structure, the covariance structure must be incorporated into the statistical model 25. Hence, following the selection of random components, a comparison of covariance structure was made in the study.

The following random effect models, which have the same fixed effects, were fitted for testing:

Model 1: Intercept, Time, Square root of time *(Random intercept and slope model)*

Model 2: Time, Square root of time *(Random slope model)*

Model 3: Time only *(Random slope model without quadratic effect)*

Model 4: Intercept only *(Random intercept model)*

All models were fitted using the REML estimation procedure, and model comparison is made using different Information Criteria. The AIC statistics show that the random intercept and slope model is the preferable model among models listed above (Table 2).

**Table 2:** Model comparison using IC for random effects using REML estimation

Random effect models	Information Criteria						
	Params	-2log	AIC	AICC	HQIC	BIC	CAIC
**Model 1**	**4**	**34392.7**	**34400.7**	**34400.7**	**34406.3**	**34414.6**	**34418.6**
Model 2	3	36567.8	36573.8	36573.8	36577.9	36584.1	36587.1
Model 3	2	39832.4	39836.4	39836.4	39839.2	39843.3	39845.3
Model 4	2	36363.7	36367.7	36367.7	36370.5	36374.6	36376.6

**Figure 5:**
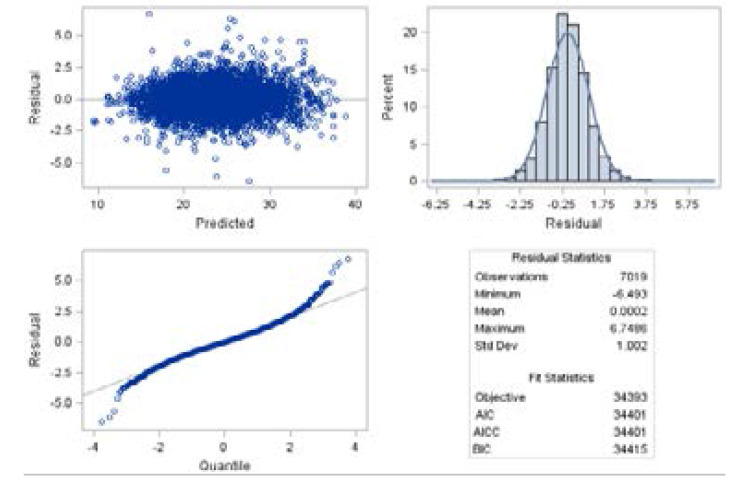
Panel of conditional studentized residuals for the square root of CD4 count

To validate the random intercept and slope model (Model 1), a panel of conditional studentized residuals for the square root CD4+ count was used. The result is presented in Figure 5. The panel consists of a scatterplot of the residuals, a histogram with normal density, a Q-Q plot, and summary statistics for the residuals and the model fit. The residuals were randomly dispersed around zero, suggesting that their mean was approximately zero. The histogram follows a normal distribution indicating a constant variance. Hence, the fulfillment of the assumption that the error term *ε_ij_* was normally distributed with mean 0 and variance *σ^i^*. Table 3 shows the comparisons between the four different covariance structures that were considered in the model using REML under the same fixed effects model. The Information Criteria was used to compare models for the structure that gives a better fit.

**Table 3:** Comparisons of covariance structure

Covariance Structure	Information Criteria						
	Params	-2log	AIC	AICC	HQIC	BIC	CAIC
AR(1)	3	35675.6	35681.6	35681.7	35685.8	35692.0	35695.0
CS	3	35671.5	35677.5	35677.5	35681.7	35687.9	35690.9
Toep	4	35671.4	35679.4	35679.4	35685.0	35693.2	35697.2
**UN**	**7**	**34087.1**	**34101.1**	**34101.1**	**34110.8**	**34125.3**	**34132.3**

The estimated unstructured covariance parameter determines the matrix (*D̂*) along with the estimated variance of the random error term (, respectively, are given below for Model 1:

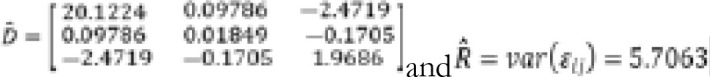


Table 4 shows the REML estimates for the fixed effects of the random intercept and slope model (Model 1).

**Table 4:** Fixed effect estimates of Model 1 for unstructured covariance structure

Effect	DF	Estimate	SE	Pr > |t|	95% C.I for Estimate
Intercept	234	24.3062	0.3055	<.0001	(23.7043, 24.9081)
Time in month	6781	0.09015	0.01072	<.0001	0.06913, 0.1112)
Sqrt_Time	6781	-0.9554	0.1036	<.0001	(-1.1586, -0.7523)
ART Initiation (Post)	195	2.4473	0.1348	<.0001	(2.1815, 2.7131)

Fitted conditional model or the subject-specific profile of the CD4+ count measure overtime ‘t’ for the two ART initiation groups can be summarized as follows:

For post ART initiation group

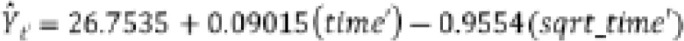


For pre ART initiation group

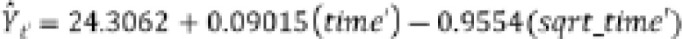


The above fitted conditional models are extended to incorporate the impact of patient's age, educational status, number of sex partners, baseline BMI, baseline viral load, and ART initiation group with the square root of CD4 count as the response. In addition to this, two-way interaction effects were evaluated within the modelling process. But, none of the interaction effects was significant. The results of the effects of age, educational status, and the number of sex partners were not found to be significant. However, we incorporate them within the modelling process since factors with subject matter importance ought to be kept within the model to eliminate any confounding effects.

The results of the fixed effect estimates are presented in Table 5. As seen from Table 5, the model intercept (*β̂*_0_) is equal to 25.2439, which is an estimate of the mean square root CD4 count at baseline (i.e., month=0) subject to other effects with covariate values set to zero in the model. The Month effect (*β̂*_1_) = 0.06377 is the slope or rate of change in the mean square root CD4 count per unit increase in the month among HIV infected patients with other covariate values set to zero. In other words, the time (month) effect shows a significant positive effect on the mean CD4 count with a rate of 0.06377 (p-value <0.0001) units per month. Hence square root CD4 count increases by 0.06377 for every month among patients, showing low progress of CD4 count over time. The effect of the square root of time (p-value < 0.0001) is also significant but appears to have an opposite effect on the square root CD4 count in a cohort of HIV infected patients enrolled in the CAPRISA 002 Acute Infection Study. The estimate for post-HAART initiation shows a highly significant positive effect with a mean square root CD4 count of 2.1104 units higher than the pre-HAART state. This implies, among patients in the post-HAART initiation group, their mean square root CD4 count increased by 2.1104, but this is not a slope. Relative to patients with normal weight status, patients with higher BMI (Obese) show a highly significant positive effect (p-value<0.0001) with 8.0201 square root CD4 count higher than the reference group (Table 5). However, underweight patients (patients with low BMI) show no significant effect compared to the reference grop. After the patients had been initiated on HAART, the average square root CD4 count among patients with a high value of the viral load at baseline is -3.2552 (p-value<0.0001) units lower compared to patients with low viral load at baseline. Moreover, after the patient had been initiated on HAART, the average square root CD4 count among patients with a medium viral load category at baseline is decreased by 1.5696 (p-value=0.0029) units compared to the average square root of CD4 count among patients with low viral load at baseline. Implying that patients with high and medium viral load at baseline have significantly lower mean CD4 count compared to patients with low viral load at baseline.

**Table 5:** Fixed effect estimates of the full Model

Covariates	Estimate	SE	Pr > |t|	95% C.I for Estimate
Intercept	25.2439	0.6040	<.0001	(24.0536, 26.4342)
Time in month	0.06377	0.009142	<.0001	(0.04585, 0.08169)
Sqrt_Time	-0.6674	0.09020	<.0001	(-0.8442, -0.4906)
ART Initiation (Post)	2.1104	0.1647	<.0001	(1.7855, 2.4353)
Baseline BMI category (ref.=Normal weight)				
Obese	8.0201	1.2896	<.0001	(5.4788, 10.5614)
Overweight	0.4966	0.5799	0.3927	(-0.6461, 1.6394)
Underweight	0.2486	0.9131	0.7856	(-1.5508, 2.0481)
Baseline HIV viral load category (ref.= Low VL )				
High VL	-3.2552	0.5633	<.0001	(-4.3652, -2.1452)
Medium VL	-1.5696	0.5211	0.0029	(-2.5965, -0.5426)
Undetectable	1.3418	3.3359	0.6879	(-5.2321, 7.9157)
Number of sex partner (ref.= Stable partner)				
Many partners	-1.4706	1.0859	0.1770	(-3.6105, 0.6693)
No partner	-0.6478	0.5791	0.2645	(-1.7889, 0.4933)
Age group (ref.= < 20)				
20–29	0.06144	0.4231	0.8847	(-0.7742, 0.8971)
30–39	0.1611	0.4780	0.7366	(-0.7831, 1.1053)
40–49	0.2491	0.6420	0.6985	(-1.0190, 1.5172)
50–59	-1.0100	1.0149	0.3212	(-3.0147, 0.9946)
≥60	-0.7631	1.9554	0.6969	(-4.6254, 3.0991)
Education attainment (ref.= Secondary or high school)				
Primary school	0.08077	1.0585	0.9392	(-2.0052, 2.1668)
Residence of participant (ref.= Urban)				
Rural	-0.2647	0.4539	0.5604	(-1.1593, 0.6298)

Spatial covariance structure measures the actual distance or variation among observations in space that are identified as unequally spaced longitudinal data 16,26. The objective of including spatial covariance structure in mixed-effects models is to account for spatial variability (heterogeneity), failure to do so can result in erroneous conclusions. The spatial covariance structure model is

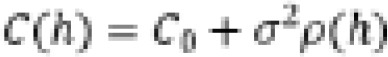

Where, *C*_0_, *σ*^2^, and *ρ*(*h*) indicates the *nugget*, the sill and the range (covariance structure model), respectively 16,26. Table 6 shows a comparison of the three commonly used spatial covariance structures: spatial exponential structure (SP(EXP)), spatial spherical structure (SP(SPH)), and spatial Gaussian structure SP(GAU). Since the exponential model has the smallest information criteria statistics and the smallest -2log suggests that the SP(EXP) structure is the best of the three spatial covariance models (Table 6).

**Table 6:** Comparison of spatial covariance models

Spatial covariance	Model Fitting Criteria						
	Params	-2log	AIC	AICC	HQIC	BIC	CAIC
SP(EXP)	9	33024.5	33042.5	33042.6	33055.1	33073.6	33082.6
SP(SPH)	9	33039.1	33057.1	33057.1	33069.6	33088.2	33097.2
SP(GAU)	9	33162.1	33180.1	33180.1	33192.7	33211.2	33220.2

The estimate of the *sill* (*σ*^2^) is 9.7063, reported as “Variance”, which corresponds to the variance of observation (Table 7). The estimated *range* (*ρ*(*h*)) is 31.1376, which appears as “SP(EXP)”, which is the practical range or distance at which the spatial autocorrelation in the exponential model is three times this amount, 3 × 31.1376 = 93.4128. That is, observations separated by more than 93.4128 distance units are not spatially correlated. In other words, the distance units indicate that observations within a participant that are close in time to be more correlated than observations farther apart in time. The estimated *nugget* (*C*_0_) is 3.4986, which appears as “Residual”, that is the value at which *h* = 0 or defined as *Intercept* in the spatial covariance structure model.

**Table 7:** Covariance Parameter Estimates of the full model

Cov Parm	Estimate	SE	Z Value	Pr>Z
UN(1,1)	3.3317	2.6772	1.24	0.1067
UN(2,1)	0.05870	0.04370	1.34	0.1792
UN(2,2)	0.004944	0.001733	2.85	0.0022
UN(3,1)	-0.3405	0.4031	-0.84	0.3983
UN(3,2)	-0.05410	0.01654	-3.27	0.0011
UN(3,3)	0.6223	0.1798	3.46	0.0003
Variance	9.7063	2.3528	4.13	<.0001
SP(EXP)	31.1376	9.4724	3.29	0.0005
Residual	3.4986	0.1008	34.70	<.0001

**Figure 6:**
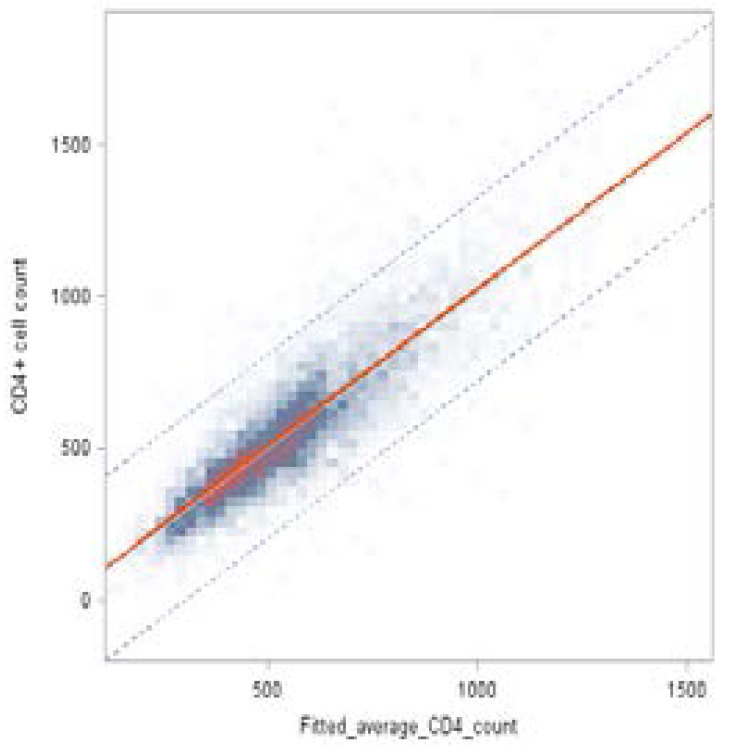
Heat map of fitted average by observed CD4 count overlaid with the fitted line

Figure 6 indic a tes the predicted profile plot for the average number of CD4+ cell, based on Table 5 results obtained by the fitted mixed-effects model. The predicted values closely matched the observed CD4+ count mean profile, with an R2=0.75, suggested that the overall model fit was good (Figure 6).

The fitted solid line in Figure 6 also indicates the estimated regression line between the observed CD4+ count and fitted values (Fitted= 148.07+0.7259 observed), and the two dashed lines show both 95% confidence interval and prediction interval.

**Figure 7:**
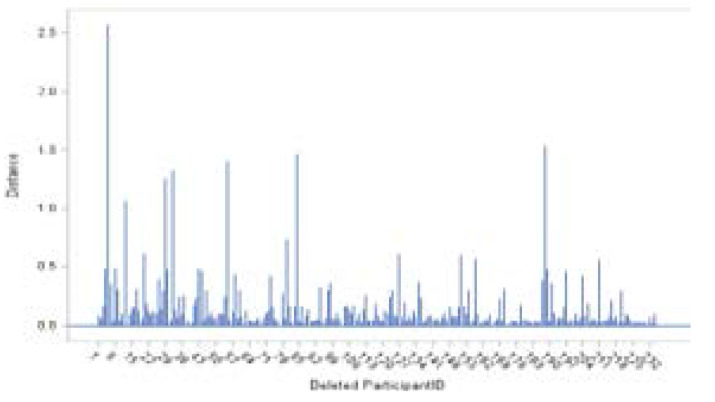
Restricted Likelihood Distance

**Figure 8:**
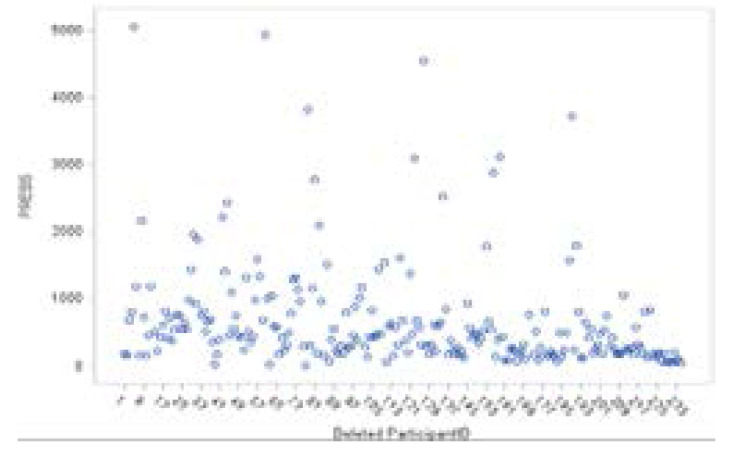
PRESS Statistics

**Figure 9:**
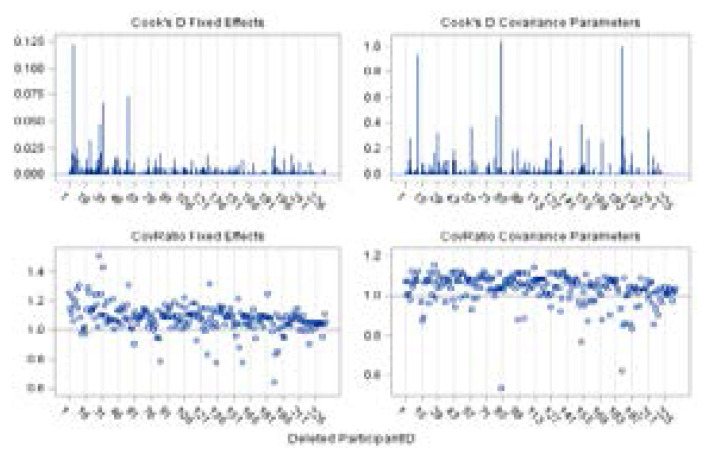
Influence statistics for the square root of CD4+ count

**Figure 10:**
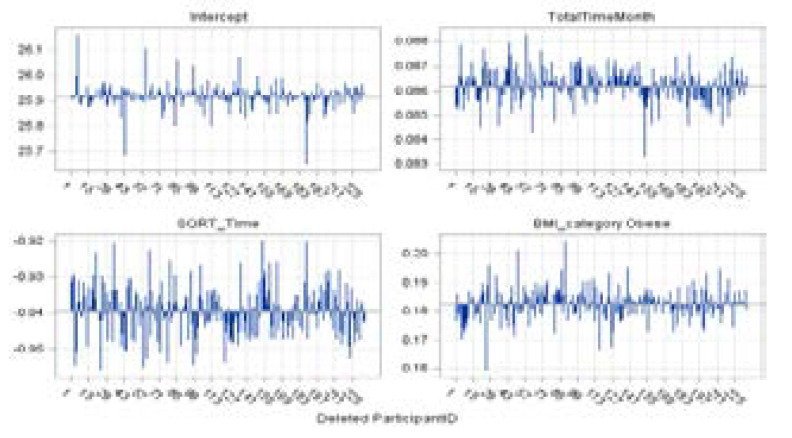
Fixed effects deletion estimates for square root of CD4+ count

**Figure 11:**
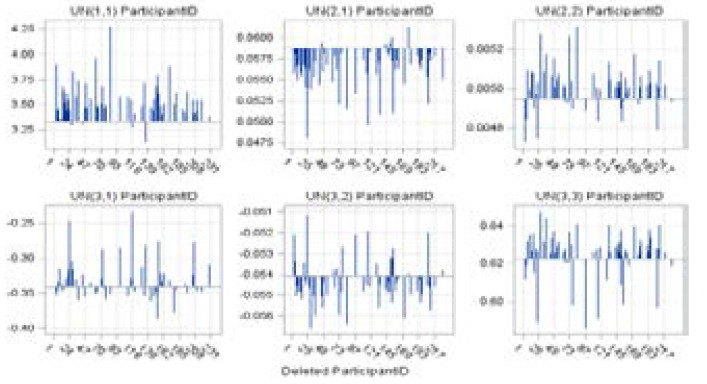
Covariance parameter deletion estimates for square root of CD4+ count

The overall influence diagnostic and diagnostics for the fixed effects are displayed graphically hereunder in Figure 7–11. Figure 7 shows the needle plot of the Restricted Likelihood Distance (RLD) for the response variable (square root of CD4+ count). The RLD plot suggests that the overall influence of patients 5, 12, 29, 32, 55, 84, and 188 stands out compared to those of the rest of the patients (Figure 7).

PRESS statistics are sums of squared PRESS residuals in the deletion sets (Schabenberger, 2005). Figure 8 shows the scatter plot of the PRESS statistics for the square root of the CD4+ count. Large values of the PRESS statistic for patients 5, 60, 84, 127, and 189 are noted.

A panel of influence statistics for fixed effects and covariance parameters is presented in Figure 9. Cook's D statistics measure the influence on the vector of parameter estimates and the CovRatio statistic measures influence on the covariance matrix of the parameter estimates. The patients with the most substantial effect on the fixed effect estimates are 5, 32, and 55 (Cook's D Fixed effects). Cook's D Covariance parameters indicate that the influence of patient 12, 84, and 188 far exceeds those of other subjects in the study data sets. This is expected since their RLD is substantial, while their impact on the fixed effects was rather moderate. The CovRatio Covariance Parameters also shows that in the absence of those patient's observations, especially patient 84 and 188, the covariance parameters may be estimated much more precisely. Note that there are other sets of observations, besides those patients listed above, that exerts influence on the chosen model (Model 1).

A panel of deletion estimates for the response variable is displayed in Figures 10 and 11 to examine how the individual parameter estimates and covariance parameters, respectively, react to the removal of the influential sets of observations^27^. Each cell in the panel (Figure 10) displays the estimates of few fixed effects that were included in the fitted model and each cell in Figure 11 displays estimates of the 3x3 variance-covariance matrix of the random coefficients and the estimate of SP(EXP) parameter following removal of sets of influential observations. Reference lines are drawn at the complete-data parameter estimates.

The focus of Figure 10 is on the behavior of individual parameter estimates that react to the removal of influential cases. Specifically, subjects 5, 44, 60, and 188 indicate a substantial impact on the model fit of the intercept. However, the removal of these subjects does not at all influence the displayed fixed effects. On the other hand, subject 27 is identified as an additional influential case since it has a strong impact on the Obese BMI category (Figure 10). Subjects 5, 29, 73, and 85 are also identified as influential cases since their presence in the data reduces the estimate of SP(EXP) parameter (Figure 11), substantially reducing the degree of correlation among data points from any patient. On the other hand, observation from subject 12 has the opposite effect. The temporal correlation drops when the impact of this patient's data is removed.

## Discussion and Conclusion

Mixed-models are one of the special statistical models that are useful in understanding longitudinal or repeated measures data. The models permit the examination of the changes over time within and between subjects. In the presence of fixed effects and random effects, the selection of an appropriate mixed model is more complicated than for a linear regression model. The fixed effect and the random effect structure are subordinate to each other, and the determination of one influences the other^28^. In this study, a step-up model selection procedure was applied to find a reasonable model that fits the data, primarily since this procedure begins with the simplest possible model and is built up by including more covariates within the model and hence does not have much numerical issue 1,18,28. In this study, the model where the intercepts and slopes were considered as random effects consolidated with the UN covariance structure was used. The results show that the prognosis of the CD4 count of a patient is significantly increased after the patient had been initiated on HAART as what we would anticipate. The impact of HIV-infected patients with the predominance of obese nutrition status (higher BMI) at baseline showed significance after patients had been initiated on HAART. Therefore, we ought to pay more consideration to the BMI of HIV-infected patients before and after HAART initiation. This may inform future techniques in studying the progression and the immunologic responses to treatment, but that does not infer that patients with higher BMI ought to be clinically ignored. Instead, based on this study and other findings, it appears that BMI contributes to some degree to drug metabolism and consequently influencing the proficiency of HAART^29^,^30^. Moreover, our results also showed that the impact of patients with higher viral load before the patient had been initiated on HAART significantly reduced their CD4 count. Therefore, effective HAART initiation after HIV exposure is necessary to suppress the increase of viral loads to induce potential ART benefits that accrue over time.

The results of the influence diagnostics analysis for the CAPRISA 002 Acute Infection study using the chosen mixed-effects model was also performed. Several cases were identified as influencing the analysis of the fitted model. Influence diagnostics analysis is essential for statistical analysis to determine how individual observations or sets of observations are influential that their presence or absence from the data impacts the analysis 31. The goal of influence analysis is not to determine observations for removal from the analysis, but to determine which cases exert undue influence on the analysis. Eliminating certain subjects from the data and base the final analysis on only the remainder is usually not the right action to take. The results of a diagnostic influence analysis can be seen only in light of the model we are working with 16.

Moreover, the data showed evidence of strong individual-specific effects on the evolution of CD4+ counts. The diagnostic plots also suggested a significant individual heterogeneity between subjects both before and after HAART initiation. Thus this may suggest that prescribing a common treatment or intervention overall patients may not be the best strategy. More research may be required to understand what factors cause patients to respond differently to treatment intervention, and such information may help to design treatment and intervention strategies that may be more efficient to a specific group of patients rather than one treatment/intervention fits all strategy.

The models depicted in this study may empower the description of the effect of several covariates on the square root CD4 count of HIV-infected patients utilizing all accessible information. We believe that this sort of analysis can be valuable to address several important issues in public health as well as offer assistance in observing patients and checking the viability of their medications. In this study, we have concentrated on the transformed normalized response data, which is the square root of CD4 count, that is continuous and conditional on the explanatory variables, and random effects have a normal distribution. Mixed models with random effects can also be applied to non-normal responses.

## Data Availability

The data used for this study can be obtained by requesting CAPRISA.

## Abbreviations

CAPRISA: Centre of the AIDS Programme of Research in South Africa; AI: Acute Infection; HIV: Human Immunodeficiency Virus; AIDS: Acquired Immune Deficiency Syndrome; CD4: Cluster of Difference 4 cell (T-lymphocyte cell); VL: Viral Load refers to the number of HIV copies in a milliliter of blood (copies/ml); ART: Antiretroviral Therapy; ARV: Antiretroviral (drug); HAART: Highly Active Antiretroviral Therapy; WHO: World Health Organization; UNAIDS: Joint United Nations Programme on HIV/AIDS; REML: Restricted Maximum Likelihood; UN: Unstructured covariance structure; MCAR: Missing Completely at Random; BMI: Body Mass Index; IC: Information Criterion.
